# Design of Metal-Based Slippery Liquid-Infused Porous Surfaces (SLIPSs) with Effective Liquid Repellency Achieved with a Femtosecond Laser

**DOI:** 10.3390/mi13081160

**Published:** 2022-07-22

**Authors:** Zheng Fang, Yang Cheng, Qing Yang, Yu Lu, Chengjun Zhang, Minjing Li, Bing Du, Xun Hou, Feng Chen

**Affiliations:** 1 School of Mechanical Engineering, Xi’an Jiaotong University, Xi’an 710049, China; fz2124@outlook.com (Z.F.); chengy@stu.xjtu.edu.cn (Y.C.); zcjun1995@stu.xjtu.edu.cn (C.Z.); liminjing92@stu.xjtu.edu.cn (M.L.); 2 State Key Laboratory for Manufacturing System Engineering and Shaanxi Key Laboratory of Photonics Technology for Information, School of Electronic Science and Engineering, Xi’an Jiaotong University, Xi’an 710049, China; zjkly19900714@126.com (Y.L.); dubing@mail.xjtu.edu.cn (B.D.); houxun@mail.xjtu.edu.cn (X.H.)

**Keywords:** SLIPS, femtosecond laser, NiTi alloy, liquid repellency, sliding performance

## Abstract

Slippery liquid-infused porous surfaces (SLIPSs) have become an effective method to provide materials with sliding performance and, thus, achieve liquid repellency, through the process of infusing lubricants into the microstructure of the surface. However, the construction of microstructures on high-strength metals is still a significant challenge. Herein, we used a femtosecond laser with a temporally shaped Bessel beam to process NiTi alloy, and created uniform porous structures with a microhole diameter of around 4 µm, in order to store and lock lubricant. In addition, as the lubricant is an important factor that can influence the sliding properties, five different lubricants were selected to prepare the SLIPSs, and were further compared in terms of their sliding behavior. The temperature cycle test and the hydraulic pressure test were implemented to characterize the durability of the samples, and different liquids were used to investigate the possible failure under complex fluid conditions. In general, the prepared SLIPSs exhibited superior liquid repellency. We believe that, in combination with a femtosecond laser, slippery liquid-infused porous surfaces are promising for applications in a wide range of areas.

## 1. Introduction

Achieving anti-adhesion and self-cleaning properties for material surfaces has attracted the extensive attention of researchers, as it can be profitable in various areas, such as in marine environments for anti-fouling [1,2,3,4,5], medical devices for anticoagulation [6,7], consumer goods for packaging [8,9,10], etc. In 2011, Wong et al. first reported a slippery liquid-infused porous surface (SLIPS) inspired by *Nepenthes* pitcher plants. Lubricants were infused into the prepared micro/nanostructures, and provided the substrates with stable and effective liquid repellency and fouling resistance [11]. Zhu et al. fabricated a type of coating with rough structures on glass, metal, and PET substrates via layer-by-layer assembly. After lubricant infusion, the surfaces showed superior sliding performance compared to various liquids. With different substrate materials used, SLIPSs can be made flexible and transparent to extend their applications [12]. Karkantonis et al. processed two types of topography on stainless steel using femtosecond laser scanning. By adopting the replica method, the structures were further prepared on polystyrene and polypropylene sheets. After infusion with silicone oil, the three substrates showed anti-adhesive characteristics when tested with liquid foods such as water, milk, and highly viscous honey [8]. Zouaghi et al. made comparisons between a conventional fluorosilane-modified superhydrophobic surface and a lubricant-infused surface in terms of fouling resistance against dairy products. The rough superhydrophobic surface failed, as nanoscale proteins could enter and become trapped inside the microscale structures, while the lubricant on the SLIPS could provide a molecular-level barrier to resist the adhesion. The protein adhesion compared to the untreated surface decreased by 63 wt.%, proving that SLIPSs are more reliable in complex environments [9,13].

Despite the great potential of SLIPSs in liquid repellency and fouling resistance, it is still a challenge to prepare SLIPSs on high-strength and hard materials such as metals. The preparation process involves three requirements [11,14,15,16,17]: (1) The lubricants should be able to wet the surface and enter the gap between rough structures. (2) The substrate should be preferentially wetted by the lubricant rather than the external liquids. (3) The lubricant and the external liquids should be immiscible. Therefore, the construction of the microstructure and the selection of lubricants become particularly crucial. Microstructure construction can be roughly classified into bottom-up (material addition) and top-down (material removal) approaches, or combined approaches [18,19]. Zhu et al. prepared a double-layer coating via a spraying method. The top layer was sprayed with a combination of graphite fluoride and PTFE to reduce the surface energy. The second layer was sprayed with a combination of α-zirconium phosphate and epoxy to help the coating adhere to the substrates. By infusion with Krytox GPL105, the SLIPS coating was formed on the steel plate [20]. Mousavi and Pitchumani et al. used an electrodeposition method on conductive copper sheets to produce multiscale structures with heights of 30–40 µm. Then, the sheets were treated with a chemical etching to induce relatively shallow textures. After low surface modification and silicone oil infusion, the prepared superhydrophobic surface and SLIPS were compared in terms of their anti-corrosion properties [21]. However, the abovementioned methods either include complex operating steps, or can only be applied to a limited range of materials. Moreover, additive coatings fabricated on some smooth substrates are likely to come off or wear away, causing the loss of slippery properties, while substrate-based microstructures do not have this problem.

A femtosecond (fs) laser, with high peak power and short pulse width, is an on-site processing method that is able to directly create precise micro/nanostructures on almost any material [22,23,24,25,26,27,28,29,30]. Moreover, the process is simple and highly controllable, making it a powerful technique for the construction of SLIPS substrates [7,31,32,33,34,35]. Herein, we converted a Gaussian laser beam to the Bessel profile, and the continuous laser pulses were transformed into pulse trains through a method of pulse shaping. The shaped laser created a uniform porous microstructure on the NiTi alloy surface, which was able to lock the lubricant after liquid infusion. Because the applied lubricant is another crucial factor that greatly affects the sliding properties of SLIPSs, five lubricants (silicone oil of three viscosities, perfluorodecalin (PFD), and perfluoropolyether (PFPE)) were chosen to infuse the laser-ablated porous surfaces and characterize their practical performances under the tested conditions, in order to study the sliding patterns and provide a reference for lubricant selection.

## 2. Materials and Methods

### 2.1. Materials

NiTi alloy substrates were received from Baoji Seabird metal material Co., Ltd. (Baoji, China), and cut into square pieces (20 mm × 20 mm). Silicone oil of different viscosities and perfluoropolyether (Fomblin^®^ Y, PFPE) were obtained from Shanghai Aladdin Biochemical Technology (Shanghai, China). Perfluorodecalin (PFD) was obtained from J&K Scientific Ltd. (Beijing, China)

### 2.2. SLIPS Fabrication

**Figure 1:**
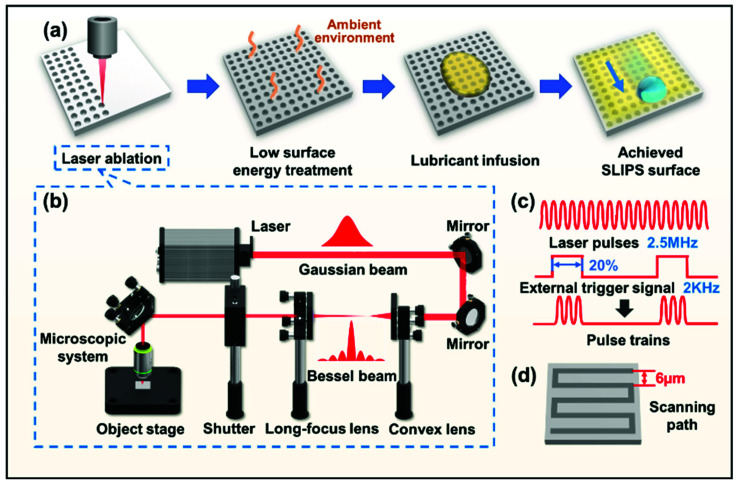
Femtosecond laser fabrication of porous microstructures on the NiTi alloy substrates: (**a**) Fabrication of the SLIPS on the NiTi alloy. (**b**) Optical path of the femtosecond laser fabrication system. (**c**) Illustration of externally triggered laser pulse trains. (**d**) Scanning path controlled by the object stage.

The process of SLIPS fabrication here involved three main steps: laser ablation, low-surface-energy treatment, and lubricant infusion, as shown in Figure 1a. Before the laser ablation, the NiTi alloy pieces were polished with sandpaper (800 mesh and 1200 mesh, in sequence) to guarantee that the surface was even in the focus point and the ablated structure was uniform. Then, all of the samples were washed in an ultrasonic bath with ethanol and water to remove dust and sand particles. The optical path of the laser system is shown in Figure 1b. During laser processing, the femtosecond (fs) laser generated by the fiber femtosecond laser system (FemtoYL-40, YSL Photonics, Wuhan, China) was controlled to be 400 fs (pulse width), 2.5 MHz (frequency), 1030 nm (wavelength), and 2.974 W (power). Then, the pulse wave generated by the signal generator functioned as an external trigger, and converted the fs laser pulses into clustered pulse trains, regulated to be 2 kHz (selecting frequency) and 20% (duty ratio). Each pulse train consisted of 250 single pulses, and the corresponding energy of an independent pulse chain was calculated to be 1487 µJ (Figure 1c). After being reflected and transported by several mirrors, the initial laser passed through a convex lens and a long-focus lens, and was shaped from a Gaussian beam into a Bessel beam. Next, the Bessel laser beam entered the microscopic system, and was focused through an objective lens (×20, NA = 0.40, Nikon, Japan) onto the metal sample, which was placed on a controllable object stage. The object stage moved regularly in line arrays at a speed of 12,000 µm/s and a scanning distance of 6 µm to match the pulse trains (Figure 1d).

After the laser processing, the NiTi alloy samples were exposed to air for 7 days to absorb carbon as a low-surface-energy treatment and, thus, achieve mild hydrophobic properties. Subsequently, different types of lubricating oil—including silicone oil of three viscosities (10 mPa·s (SiO-10), 200 mPa·s (SiO-200), and 500 mPa·s (SiO-500)), PFD, and PFPE—were infused into the treated samples to fill the porous structures and form a thin oil layer on the surface, endowing the surface with sliding properties.

### 2.3. Characterization

Contact angles (CAs) and sliding angles (SAs), as well as the sliding performances of the tested liquids on the prepared SLIPSs, were characterized using a contact angle meter (JC-2000D, Powereach, Shanghai, China). The droplet adhesion forces of deionized water and ethanol were tested with a surface tension meter (DCAT11, DataPhysics, Filderstadt, Germany). The laser-ablated porous structure was observed with a scanning electron microscope (SEM, Flex1000, Hitachi, Tokyo, Japan). In the durability test, high-temperature cycling was carried out in a heating chamber (KCSHWHS, AISRY, China), and the hydraulic pressure test was carried out in an industrial pressurized cabin (YX-JL-QF30-40L, YUEXIN, Guangzhou, China).

## 3. Results and Discussion

### 3.1. SLIPSs on the NiTi Alloy

**Figure 2:**
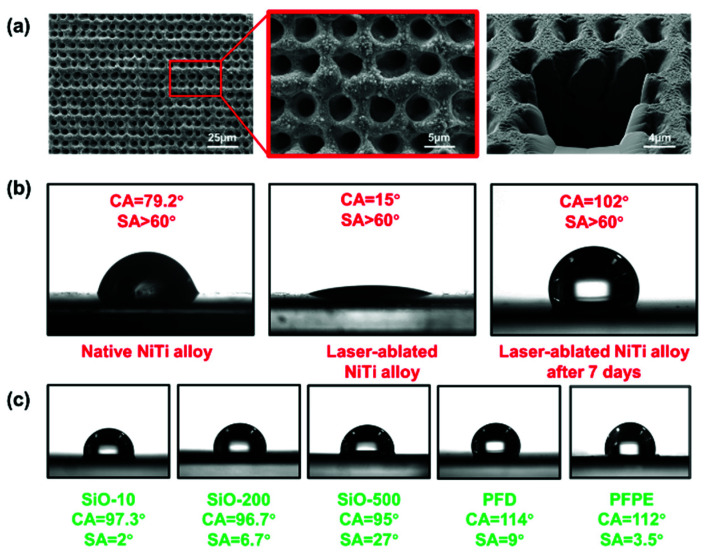
Microstructure of the NiTi alloy surface and its water-repellent properties: (**a**) Top view and cross-sectional view of the structure taken by scanning electron microscope. (**b**) Wettability of the native NiTi alloy samples after laser ablation and after being placed in air for 7 days. (**c**) Wettability of the SLIPSs prepared using SiO-10, SiO-200, SiO-500, PFD, and PFPE.

The fabrication of SLIPSs includes structure construction on substrates and the infusion of lubricants (Figure 1a). An appropriately rough structure is one of the essential criteria in preparing SLIPSs. Herein, the fs laser pulse was triggered by an external signal and temporally divided into pulse trains. Through adjusting the object stage with the corresponding running speed and scanning line distance, laser pulse trains were able to match with the running path and, thus, ablate the ordered and uniform structure. In addition, as the Bessel beam had a large depth of field and small spot size, it showed a specific advantage of processing porous structures with a high depth-to-diameter ratio on metal surfaces. As shown in the optical path in Figure 1b, the fiber femtosecond laser system output the infrared Gaussian beam, and it was subsequently shaped into a Bessel beam by a convex lens and a long-focus lens, increasing the depth of field and reducing the spot size. Therefore, the energy of each pulse train was absorbed by the NiTi alloy and, accordingly, created a deep microhole on the surface. SEM images of the top view and cross-sectional view show the details of the laser-ablated structure from the surface and the inside (Figure 2a). The porous structure at a micro scale was neatly distributed on the NiTi alloy, with a spacing of around 6 µm. The distribution period can be controlled by the scanning path and the laser frequency, as mentioned above. The microhole diameter was about 4 µm, as a result of the laser power and the spot size of the Bessel beam. Meanwhile, a large number of nanoparticles could be seen scattered around the microholes, because the material was molten, sputtered, and then solidified on the surface during processing caused by the high peak power of the fs laser. The cross-sectional view showed the vertical depth and the spatial pattern of the microporous structure. The microholes featured a depth of about 6 µm, and the shape of the internal channels sloped irregularly at the interior of the NiTi alloy, which could be attributed to the adopted Bessel beam. This special structure can be filled with sufficient lubricant and provide beneficial lubricant storage conditions.

The general process of SLIPS fabrication on a laser-ablated surface is shown in Figure 1a. After cleaning the surface, the native NiTi alloy exhibited a hydrophilic property, with an intrinsic CA of 79.2° (Figure 2b). Its SA exceeded 60°, and the water droplet was unable to slide off the surface. After fs laser ablation, the CA was reduced to 16°. According to the Cassie–Baxter model, the microstructure on the surface can amplify its wettability, making the hydrophilic surface more hydrophilic, and vice versa. As the SLIPS substrate should be preferentially wetted by the lubricant, a low-surface-energy treatment was required here. Instead of using chemical modification, which is more complex and causes environmental pollution, we simply placed the samples in the ambient environment, and the laser-ablated NiTi alloy surfaces were able to absorb the element carbon in the atmosphere, resulting in the CA increasing to 102°, although it still showed poor sliding performance (SA > 60°). Then, the samples were dispensed with lubricants and placed in a vacuum environment for 5 min to help the oil penetrate into the microporous structure and become locked in the pore channels.

Five types of lubricants—including silicone oil of three viscosities (10 mPa·s, 200 mPa·s, and 500 mPa·s), PFD, and PFPE—were adopted, and we further investigated their water sliding performance (Figure 2c). Among the silicone-oil-infused samples, their static CAs were around 96°, and did not show significant fluctuation, as the surface energy of silicone oil with different viscosities is generally about 21 mN/m. However, the SA increased as the viscosity became higher. The silicone oil of lower viscosity, featuring a relatively higher fluidity, showed a smaller sliding resistance on the top layer, so the droplets were likely to be more affected by the gravity and slide off the surface. As for the PFD- and PFPE-infused substrates, they showed a higher CA (114° and 112°, respectively), as the -CF3 functional group has a lower surface energy [36]. The SAs of most prepared samples were below 10°, except for the SLIPS with SiO-500 (SA = 27°), proving that the applied lubricants remarkably improved the sliding performance of the NiTi alloy.

### 3.2. Droplet Adhesion Force

**Figure 3:**
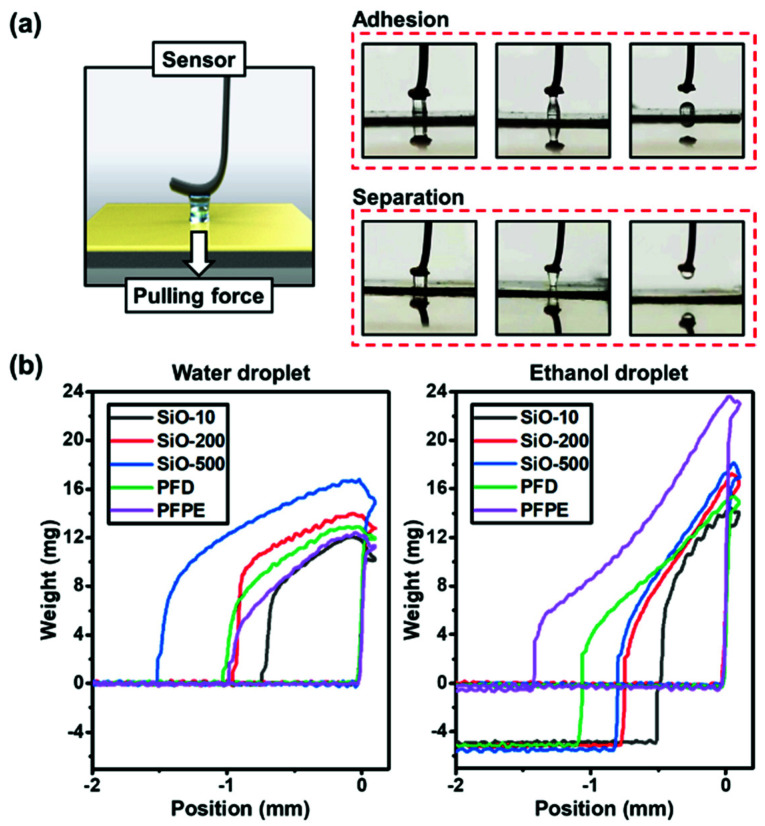
Droplet adhesion force tested with water and ethanol: (**a**) Adhesion and separation occurred between the droplet and the surface. (**b**) Droplet adhesion of water and ethanol on the prepared samples tested using high-precision sensors.

Although SLIPSs display self-cleaning ability by encouraging the droplets to slide off, partial adhesion or even droplet pinning may occur, as there is a possibility of fast-moving liquids accidentally touching the substrate. Because the liquid-repellent properties of SLIPSs are determined by the lubricants applied on the surface, herein, the droplet adhesion force between the lubricating layers and the droplets was measured to evaluate the immiscibility of the samples with external liquids. The test was performed on a surface tension meter. Deionized water and ethanol were chosen as the tested liquids. A droplet (volume = 10 µL) was dripped onto the tip of the probe, which was fixed on a moving motor equipped with a high-precision sensor. With the motor controlling the droplet to vertically descend, reach, and then leave the sample surface, the sensor was able to detect the adhesion force added to the droplet’s weight and record the change in the pulling force, as shown in Figure 3a.

Two states of droplets could be observed during the experiment: In the first case, the droplet on the probe was torn apart, and part of it was stuck to the surface due to excessive adhesion force while leaving. The surface displayed poor liquid repellency, as presented in the line graph where the measured weight value dropped below 0 mg, indicating a decrease in the droplet size compared to the initial value, i.e., “adhesion” (Figure 3b). In the other case, the droplet received less adhesion force and was able to be detached from the sample surface intact, exhibiting a high degree of immiscibility for the large surface energy difference between the lubricating layer and the droplet, i.e., “separation” occurred. In the water droplet adhesion test, all SLIPSs maintained good performance, and no water droplets were stuck to the samples. More specifically, silicone-oil-infused samples with lower viscosity had less adhesion, and the perfluorinated lubricants PFD and PFPE manifested the lowest adhesion force of all. The situation changed when the test was performed with ethanol. The PFPE-infused sample was still able to separate the droplet from the surface and keep the weight unchanged, while the other samples failed to some extent. As can be seen in the graph (Figure 3b), the droplet weights fell to negative values when the probe returned to its original position. This is because ethanol has a lower surface tension (21.9 mN/m) compared to water (72.8 mN/m), making it easier for it to spread out and stick to the surface [37]. As for the applied lubricants, substances with lower surface energy—such as PFPE—have a relatively wider range of liquid resistance, and make the SLIPS less likely to be subject to droplet pinning, reducing the risk of failure (especially under dynamic conditions).

### 3.3. Droplet Sliding Performance

**Figure 4:**
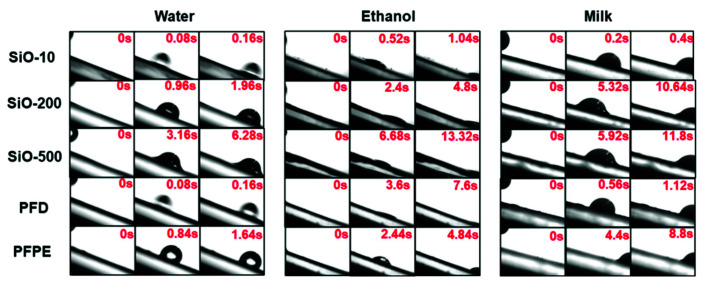
Sliding process and time spent for three liquids (water, ethanol, and milk) on the lubricant-infused samples.

Since the static droplets were adhered to the SLIPSs in the droplet adhesion force test, the sliding performance with different liquids was further characterized. The prepared SLIPSs were tilted at 20°, and an equal volume of droplets was dripped onto the sample surfaces. The CCD recorded the droplets from entering to leaving the field of view at 25 fps, and the images were analyzed to compare the total sliding time of the movement. As shown in Figure 4, the time increased with rising viscosity in the silicone-oil-infused samples, while most of the SLIPSs effectively allowed the droplets to slide quickly within 2 s. Even SiO-500 showed a decent performance, with time = 6.28 s. As for ethanol, due to its much lower surface tension, it can be clearly seen that the droplets spread out on the surface, maintaining a very low CA, but could still fall off the tilted surface, with times ranging from 1 s to 13 s. In terms of milk, which contains a complex composition and can be seen as common liquid food in daily life, the prepared surfaces also exhibited excellent sliding properties to keep themselves uncontaminated, even though the sliding time fluctuated to a great extent with the different lubricants used (0.4 s~11.8 s). All of the prepared samples effectively allowed different liquids to slide off. It can be concluded that SLIPSs have the ability to repel various kinds of liquids, even if the droplets exhibit temporary affinity for the lubricating layer. Therefore, although SLIPSs have the risk of being adhered to by droplets—especially low-surface-tension liquids—they still possess unique sliding properties, and allow droplets to slide off the surface at certain angles, giving the substrates a liquid-repellent ability.

### 3.4. Durability Test

**Figure 5:**
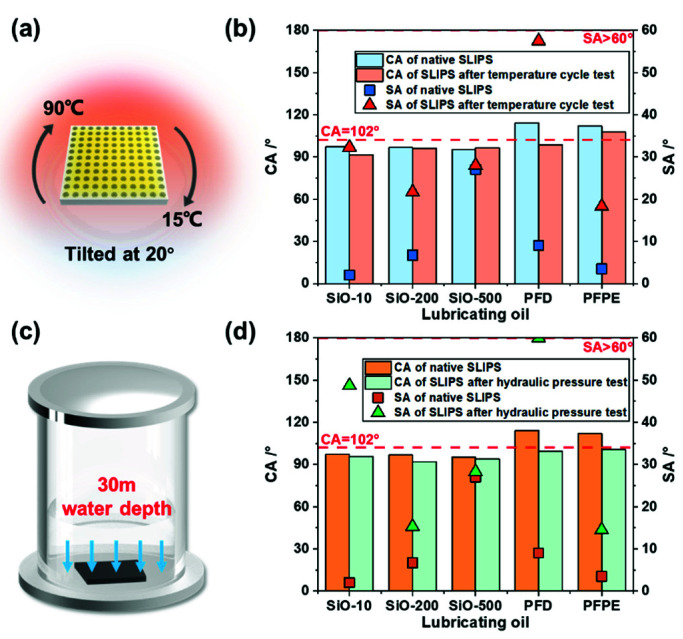
Durability test implemented on the SLIPS samples: (**a**–**d**) CA and SA of five lubricant-infused surfaces before and after the temperature cycle test and the hydraulic pressure test. The CA and SA of the NiTi alloy after 7 days are marked by the red lines.

SLIPSs have potential applications in the fields of aircraft, aerospace, marine transportation, etc., which often involve harsh environments. Therefore, durability tests were conducted, including the temperature cycle test and the hydrostatic test. A heated chamber was used to perform the high-temperature cycle test. SLIPS samples were tilted at 20° and placed in the chamber, and the temperature was changed every 2 h between 15 °C and 90 °C, for a total of 48 h, i.e., 24 temperature cycles (Figure 5a). As shown in Figure 5b, the CAs did not fluctuate very much among the silicone oil samples, while the change in their SAs after the test increased as the viscosities became lower. In particular, the SA of the SiO-10 sample increased greatly from 2° to 32.3°, while the SA of the SiO-500 sample remained stable. Under the influence of the 20° inclined surfaces, the low-viscosity lubricant, while providing good sliding properties, was also more likely to be lost along the surface—especially in the presence of external contaminants sliding through during practical use. Moreover, it is noteworthy that the most significant change after the test was for the PFD-infused surface. Through observing the surface, it was found that the lubricant had completely evaporated and none remained. Its wettability was close to that of the NiTi alloy after 7 days (marked by the red lines), and failure occurred, indicating that the volatility of the lubricant was another main reason for the failure of the SLIPS. The other three groups were relatively stable in terms of their tested values (SA = 20°~30°), and maintained decent sliding properties, showing that the SLIPSs kept high-temperature stability in general.

The hydraulic pressure test was further carried out on the SLIPSs. The samples were placed in an industrial pressurized cabin for 18 h in a simulated water depth of 30 m (approximately 300 MPa), in order to assess their resistance to water pressure (Figure 5c). The results are shown in Figure 5d. Samples infused with SiO-10 (SA = 48.7°) and PFD (SA > 60°) lost their sliding performance during the test. Lubricants with lower viscosity, such as SiO-10, were prone to being extruded from the microstructure by high water pressure because of their higher fluidity, and since the density of the overflowed silicone oil (0.96 kg/m^3^) is slightly lower than that of water, it subsequently floated to the water surface, causing the loss of the lubricant. In conclusion, the SLIPSs with low-viscosity silicone oil can provide excellent sliding properties, while those prepared with high-viscosity oil show better durability in general. Moreover, the SLIPS with PFD experienced failure because of its volatile nature, and its wettability returned to the intrinsic value of the substrate. The remaining three samples showed relatively small changes in SA, and maintained good sliding performance after the test (SA of SiO-200 and PFPE < 20°; SA of SiO-500 = 28.3°). Therefore, the SLIPSs treated with SiO-200, SiO-500, and PFPE have the ability to resist high water pressure.

## 4. Conclusions

In this paper, we used a femtosecond laser to fabricate porous microstructures on NiTi alloy surfaces. Though adapting the proper laser parameters with the running path of the object stage, microscale holes were uniformly created on the surface, providing beneficial lubricant storage conditions for the SLIPSs’ construction. After infusing the NiTi alloy substrate with lubricant, the surfaces were endowed with excellent sliding properties, with most of the sample surfaces having an SA of less than 10°. Meanwhile, to maximize the performance, comparisons between lubricants as slippery substances were made for different applications. Five different lubricants—including silicone oil of three viscosities, PFD, and PFPE—were used to prepare SLIPSs, and a series of tests were conducted to quantify their sliding behaviors. It was found that relatively low-viscosity silicone oil had superior sliding performance, while high-viscosity silicone oil showed more stable durability in the temperature cycle test and the hydraulic pressure test. Additionally, it was found that samples infused with PFD failed easily because of its highly volatile nature. In addition, water and ethanol were used to characterize the prepared samples for their ability to repel multiple liquids. Relatively, the perfluorinated lubricants PFD and PFPE showed better hydrophobic properties for the tested liquids, due to their low surface energy, while all of the prepared samples presented outstanding sliding properties for different external liquids, achieving the function of liquid repellency on the surface. Combined with the femtosecond laser preparation method, slippery liquid-infused porous surfaces are expected to be prepared on more material substrates and used in more extensive fields.
